# Spiral magnetic domain structure in cylindrically-shaped microwire**s**

**DOI:** 10.1038/s41598-018-33322-0

**Published:** 2018-10-10

**Authors:** A. Chizhik, A. Zhukov, J. Gonzalez, P. Gawroński, K. Kułakowski, A. Stupakiewicz

**Affiliations:** 1 Departamento de Fisica de Materiales, Universidad del País Vasco, UPV/EHU, San Sebastian, Spain; 2 IKERBASQUE, Basque Foundation for Science, 48011 Bilbao, Spain; 3 AGH University of Science and Technology, Faculty of Physics and Applied Computer Science, 30-059 Krakow, Poland; 4 Faculty of Physics, University of Bialystok, 15-245 Bialystok, Poland

## Abstract

Identification and characterisation of novel and unusual magnetization states remains a topic of research in modern magnetism. Recently, control of the magnetization state between the surface and volume in cylindrical microwires with the giant magneto-impedance effect has been demonstrated. Herein, the phenomenon of spatial migration of spiral magnetic domains inside a microwire is demonstrated using the magneto-optical Kerr effect. The main properties of the inclined spiral structure were determined, where the surface domain structure possessed a length limited only by actual sample length. Transformation of the structure from a spiral to an elliptical structure could be controlled by external torsion stress. Hysteresis and magnetic images were simulated based on a model assuming a spatial distribution of the internal stress inside the microwire, whose results were consistent with the experimental results. A consistent interpretation of the results in terms of the formation and transformation of the spiral magnetic domain structure is proposed.

## Introduction

Magnetic amorphous glass-covered microwires have demonstrated a wide variety of domain structure types and magnetization reversal scenarios^1,2^. Further, prediction and control of the magnetic domain structure has been accomplished by varying the microwire composition, thermal treatment conditions, external stresses and the combination of magnetic fields in microwires^3–5^. Recently, we have reported a technological course that permits fine tuning of a domain structure^6^, which consists of the selection of thermal treatments, torsion and tensile stress applications, and the capability of DC, AC, axial and circular magnetic field combinations. Determination and classification of the maximum number of domain structures is an important task because of the role that the domain structure plays in the functioning of different types of microwire-based technological elements. The magnetic structure of a microwire consists of an axially magnetized inner core and an outer shell whose magnetization depends on the parameter series. In particular, the outer shell can exhibit mono-domain or multi-domain structures. Radial and circular domains are the main domain types found in the outer shell. Further, under certain conditions, the circular domain structure is transformed into helical domain structures with different angles of helicity.

In the search for new types of domain structures, we have focused on the so-called spiral domain structure. The in-plane spiral domain structure was first reported in the 1980s^7^, and herein we report an in-volume spiral domain structure experimentally identified in a magnetic microwire. The cylindrical shape of the microwire is the key property that promotes the in-volume spiral domain. The essential property of this type of domain structure that associates it with the in-plane spiral structure is the extremely long domain walls (DWs). Simultaneously, the non-planar nature of the spiral structure in the microwire exhibited further interesting features.

## Results

### Sample characteristics and geometry

We studied stress-annealed glass-coated microwires with the Fe_71.7_B_13.4_Si_11_Nb_3_Ni_0.9_ composition^6,8,9^, a metallic nucleus diameter of *d* = 103 µm and a total diameter including the glass coating of *D* = 158 µm. The microwires were prepared by the Taylor-Ulitovsky method previously described elsewhere^10,11^. Sample annealing was performed in a conventional furnace at temperatures, *T*_ann_, below the crystallization temperature and the Curie temperature such that *T*_ann_ ≤ 300 °C. The thermal treatment could be performed in air because the metallic nucleus was coated by a continuous insulating glass coating. The microwire was heated, annealed and slowly cooled within the furnace while under tensile stress.

### Experimental demonstration

Imaging of the magnetic domains and hysteresis loops on the surface of microwires were performed via optical polarizing microscopy in the reflective mode using the longitudinal magneto-optical Kerr effect (MOKE) geometry^12^. A mechanical torsion stress *τ* was applied to the studied microwire, whereby one of the wire ends was mechanically fixed and other end was rotationally stressed to apply the torsion.

**Figure 1:**
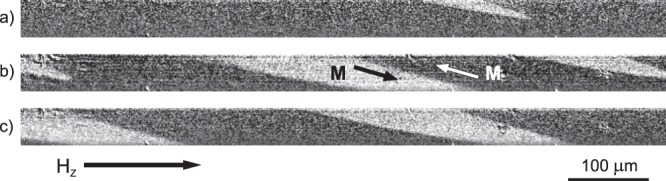
MOKE images of the spiral magnetic domain structure formation at various axial magnetic field, *H*_Z_, values. (**a**–**c**) *H*_Z_ values of (**a**) 10, (**b**) 20, and (**c**) 30 A/m. The black and white areas correspond to domains with different orientations of the magnetization (*M*), whose directions are marked by arrows.

The formation and transformation of the surface spiral domain structure in the presence of an axial magnetic field (*H*_Z_) is shown in the Fig. 1. The magnetization reversal process began with the formation of wedge-like inclined domains (Fig. 1(a)), and at the second stage the wedge-like domains moved along the wire surface in such a way as to form a quasi-periodic structure.

**Figure 2:**
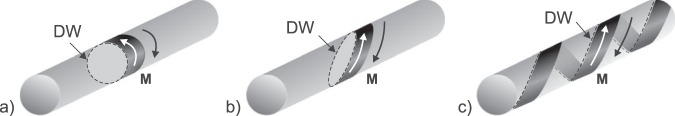
Schematic of the different types of magnetic domain structures existing in microwires. (**a**) Circular magnetic domains, (**b**) elliptically-shaped magnetic domains, (**c**) and spiral magnetic domains. The orientation of the magnetization in the domains (arrows) and the domain walls (dashed lines) are indicated.

As the domain area increased, a subsequent collective motion of the entire domain structure along the wire was observed. The main property of the observed structure that should be especially emphasized is the long length of the domains and, correspondingly, the DWs. Figure 2 schematically illustrates the different types of magnetic domain structures that are intensively studied in amorphous microwires.

The length of the DWs is the main difference between domain structure observed herein and the previously-reported circular^1^ (see Fig. 2(a)) and inclined elliptic helical structures^13^ (see Fig. 2(b)). Specifically, herein we observe a drastically different structure with a long length (see schematic in Fig. 2(c)).

**Figure 3:**
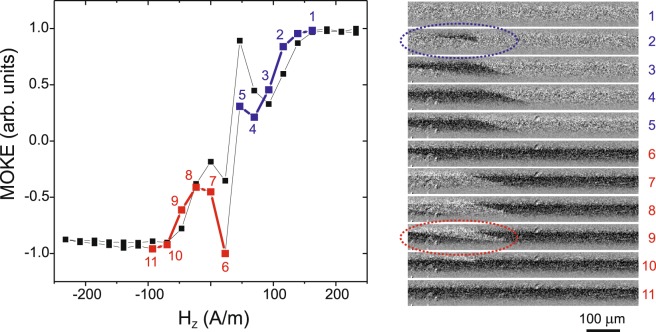
MOKE hysteresis loop (left panel) and images (right panel) obtained using a MOKE microscope in the presence of the torsion stress *τ* = 10 π rad∙m^−1^ in the microwire through the application of a magnetic field *H*_Z_. The numbers (1–11) (left panel) correspond to the domain image numbering in the right panel.

It has been observed in the case of the inclined helical structure that the spiral structure is very sensitive to an external mechanical torsion stress. Herein, therefore, we applied a torsion stress and revealed a transformation of the magnetization reversal process that was realized in the transformation of the hysteresis loops and the surface domain structure (Fig. 3).

Figure 3 presents images of the domain structure that correspond to points in the plotted hysteresis loop, correlated by the numbers 1–11. The presence of a torsion stress increased the complexity of the magnetization reversal process, which comprised three definite regions. The first two regions, respectively marked in blue and red colours in the hysteresis loop plot (Fig. 3), correspond to the formation of black and white wedge-shaped spiral domains (marked by the coloured dashed circles in Fig. 3). The third region corresponds to a very short process comprising a rapid jump between images 5 and 6 in Fig. 3. This process is similar to the previously-observed process of helical bistability^1^, where herein we observe the local manifestation of this effect. Therefore, we posit that the torsion stress incompletely suppresses the formation of the spiral structure, while simultaneously the presence of the external stress induces the formation of spiral structures possessing different magnetization directions within the magnetization reversal cycle.

### Simulations

A theoretical analysis of the obtained results was performed on the basis of the assumption that, for a magnetostrictive microwire, the cylindrical symmetry of the wire limits the stress to radial (*srr*), circumferential (*sqq*) and axial (*s*zz) components (i.e., a different stress along each axis). Further, the energy of the magnetic anisotropy is considered equivalent to the energy necessary to rotate a local magnetic moment. In other words, the magnetic anisotropy energy in the wire is assumed to possess a uniaxial character equivalent to a cylindrical symmetry of the angular dependence of the energy of the local magnetic moments. That is to say, for a local easy axis along the *Z* direction, the energies needed to rotate the magnetic moment within the *ZX* plane and within the *ZY* plane are the same. The concept of an effective uniaxial anisotropy, *K*_U_, considers that the magnetic moment is rotated within the plane where the energy cost is lowest. Thus, if the circumferential, radial and axial magnetic moment component values rank as *σ*_*qq*_ < *σ*_*rr*_ < *σ*_*zz*_, then *K*_U_ = 3*λ*(*σ*_*zz*_−*σ*_*rr*_)/2, where *λ* is the magnetostriction constant. Thus, the energy to rotate the magnetic moment in the *ZX* plane is less than that necessary to rotate it in the *ZY* plane^14^.

**Figure 4:**
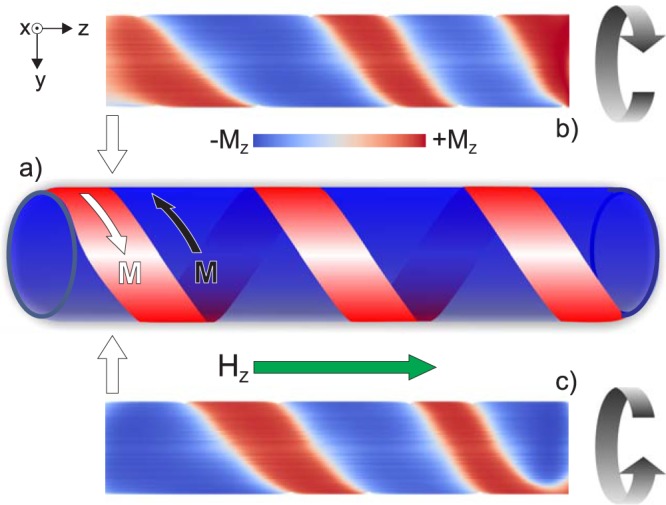
Images of the simulated surface domains demonstrate the spiral nature of the observed structure. (**a**) Schematic of the spiral domain structure. (**b**,**c**) Simulated domain structure of the (**b**) front and (**c**) back part of the microwire.

Using the simulations, first we demonstrated the spiral character of the obtained structure (Fig. 4), showing the simulated domain structure obtained on two opposite surface sides of the microwire (i.e., front and back). These images clearly exhibit a structure radically different from the inclined ellipse helical structure wherein the domain structure from the back side exhibited an opposite direction of the inclination^15^.

**Figure 5:**
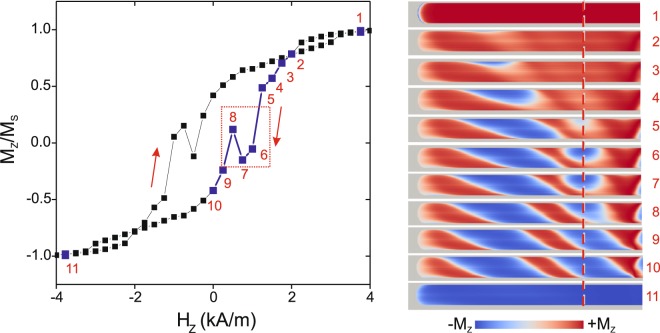
Simulated hysteresis loops as the normalized magnetization (*M*_Z_/*M*_S_) (left panel) and domain images (right panel) obtained by simulations of the microwire in an applied magnetic field *H*_Z_. The numbered points in the hysteresis loop (points 1–11 in the left panel) correspond to the numbered domain image (right panel).

The simulated magnetization reversal process is given in Fig. 5, plotting the local hysteresis loop obtained from a single spot 20 × 20 × 40 nm^3^ in dimension and 5.25 µm from the edge of the microwire (red dashed line in Fig. 5 right panel images). The shape of the simulated hysteresis is consistent with the experimental MOKE hysteresis (Fig. 3), where the essential element observed in both the experimental and simulated loops is the local part (red dashed box in Fig. 5).

Comparing the simulated hysteresis loop plot and the simulated images, it can be concluded that this local part is related to the formation and transformation of the wedge-like spiral structure, as was true for the experimental hysteresis loops and images herein.

A local decrease of the relation between the areas with a magnetization parallel and anti-parallel to the external axial magnetic field (red and blue domains in Fig. 5, respectively) is observed in the short range of the *H*_Z_ variation. Following the general trend of magnetization reversal, the mono-domain (blue domain in Fig. 5) transforms in a consistent manner to the anti-parallel mono-domain (red domain in Fig. 5), which is reflected in the hysteresis loop shape in Fig. 5. Further, the spiral character of the observed domains causes a local shift and a subsequent local decrease of the domain area at the fixed region the calculation was performed. This local consideration corresponds completely with the MOKE experiments, where the MOKE signal was obtained from a local region of the wire sample and not from the entire wire. The simulated hysteresis, however, does not contain the second local part observed in the MOKE experiment, but we consider this a nonessential difference.

**Figure 6:**
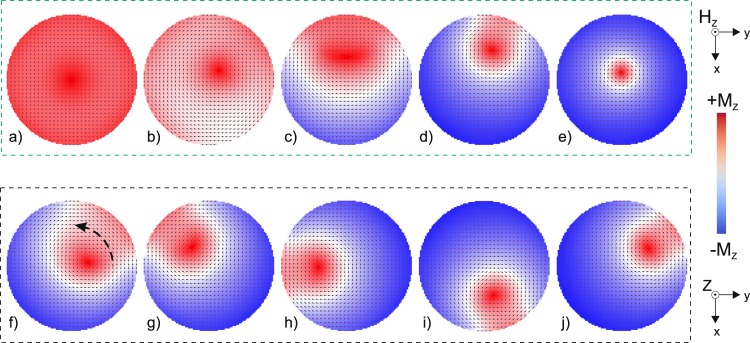
Cross-sections of the simulated magnetic structure in a microwire (**a**–**e**) at a fixed *Z* = 5.25 µm and varying *H*_Z_ values of (**a**) 2.25, (**b**) 1.25, (**c**) 0, (**d**) −2.25, and (**e**) −3.25 kA/m; and (**f**–**j**) at a fixed *H*_Z_ = −2.5 kA/m and varying distance *Z* from the edge of the microwire of (**f**) 0.6, (**g**) 1.8, (**h**) 2.4, (**i**) 3.6, and (**j**) 4.8 µm. The arrow in (**f**) shows the spiral domain migration direction inside the microwire. The colours correspond to a magnetization with +*M*_Z_ (red) and −*M*_Z_ (blue) orientations.

To gain a deeper understanding of the magnetization reversal with spiral domains, transverse cross-sectional images of the simulated magnetic structure were obtained. These simulations were obtained for varying axial magnetic field at a fixed distance of 5.25 µm from the edge of the microwire (Fig. 6(a–e)) and for varying locations along the length of the microwire at a fixed axial magnetic field of −2.5 kA/m (Fig. 6(f–j)).

As expected, in the presence of the axial magnetic field, the magnetization reversal begins from a saturated magnetic state (red colour in Fig. 6(a)). The surface of the microwire is the natural nucleation source for the reversed magnetic state. Thus, as the axial magnetic field is increased the inclination of the magnetization originates from the surface and leaves in the centre in a partially saturated state (Fig. 6(b)). With further increase in the magnetic field, a reversed domain is formed in the surface area, shifting the original magnetic state to the opposite side of the cross-section (Fig. 6(c)). It is at this stage that the formation of the pronounced spiral structure can occur. Further increase of the magnetic field causes the visible location of a spiral domain and a decrease of its volume (Fig. 6(d)). At the final stage, the spiral domain disappears and the only un-reversed state left in the cross-section remains in the core of the microwire (Fig. 6(e)).

Figure 6(f–j) show the cross-sections of the magnetic structure of the microwire obtained at different distances *Z* along the length of the wire in a fixed axial magnetic field *H*_Z_ = −2.5 kA/m. The nucleus of the spiral domain appeared inside the wire (darker red area in Fig. 6(f)), where the position of the nucleus was asymmetric relative to the microwire core. The blue dashed arrow in Fig. 6(f) shows the ordered migration of the reversed red domain inside the wire, and the full series of images confirm the spiral and unlimited character of the observed domain structure.

In the case of circular domains^8^, the periodicity of the domain structure is determined by the interplay of the domain nucleation and the DW mobility. For the case of spiral domain structure, however, the magnetization reversal consists of the nucleation of a solitary inclined wedged domain and its subsequent movement along the surface of the microwire. The torsion stress is the interference of two tension stresses of opposite signs^16^ (i.e., expansion and compression) along the two directions inclined 45° from the axis of the microwire. An increase of the torsion stress changes the proportion between the expansion and compression, which induces a change of the orientation of the surface magnetization owing to the magnetoelastic interaction. This, in turn, causes the change of the periodicity of the spiral domain structure.

## Discussion

We consider that, in the case of a spiral domain structure, the traditional model of “inner core–outer shell” discussed earlier may be subject to reconsideration. Herein, we observe a partially-magnetized spiral domain whose location migrates depending on the distance along the length of the microwire. Examined from another perspective, at some stages of the magnetization reversal the spiral domain could be considered a reduced outer shell when taking the surface location of the domain into account. The narrow localization of the spiral domain and its weak correlation with the volume magnetic state are the evident causes of the high mobility of the structure experimentally observed herein.

The spiral nature of this studied structure dictates the long length of the domains and long unclosed structure of the DWs, which are limited only by the length of the microwire. Generally speaking, a long-range interaction between greatly-separated sample points may establish a long DW. However, a spiral DW establishes a long-distance correlation between greatly-separated sample points. Under these conditions, the spiral DW is the key factor for the fast magnetization reversal.

The influence of the torsion stress is also an important factor. As observed in the MOKE experiment, the magnetization reversal in the presence of a torsion stress included two different mechanisms related respectively to the spiral and elliptical structure (Fig. 3), where suppressing the spiral structure stress supported the elliptical structure. Though these two structures are somewhat similar in that they both exhibit a helical nature with varying angle of helicity, they possess an essential difference wherein the elliptical structure has a closed DW while the DW of the spiral structure extends infinitely. The infinite nature of the spiral structure is related to the axial internal stress, while the torsion stress induces an additional circular internal stress that induces the formation of the circular or elliptical domain structure.

In addition, we were able to obtain magneto-optical imaging of the opposite sides of the microwire^15^, whereby the transformation of the surface domain structure image was observed as the focus distance of the objective was changed. With this method, the change of the direction of the inclination of the domain structure was not observed, which is evidence of a spiral structure. The torsion stress induces a transformation of the magnetization reversal and the domain structure in the wire as a whole, but not at the surface alone. An increase of the torsion stress forms a structure that is sufficiently homogeneous in the entire volume of the microwire, including the surface region^13^.

## Conclusions

A novel spiral domain structure was found in a magnetic microwire, and further theoretical calculations confirmed the feasibility of the existence of this type of magnetic structure. The main stages of the magnetic field-induced hysteresis of this domain structure were determined and analysed. Magnetization reversal began from a wedge-like structure that transformed to a spiral structure. The quasi-periodicity of the spiral structure changed during magnetization reversal, which was reflected in a characteristic local anomaly observed in the experimental and theoretical hysteresis loops. Analysis of the simulated magnetic structure cross-sections supported an extension of the “inner core–outer shell” model by adding the statement that the spiral domain structure can alternately play the role of the local inner core or the local outer shell.

An external torsion stress induced a variation of the type of surface magnetic structure by suppressing the spiral structure and enhancing the elliptical structure, where the decisive factor for the expressed structure was the internal stress distribution inside the microwire.

## Methods

### Magneto-optical Kerr effect technique

Observation of the magnetic domains on the surface of glass-covered microwires was accomplished using magneto-optical polarizing microscopy working in the reflective mode and using the longitudinal MOKE configuration^12^. The surface magnetic domains were visualized owing to the different in-plane components of the magnetization that transformed to a black-white magnetic contrast when the polarized light was reflected from the cylindrical surface of the microwire. The obtained MOKE images of the domain structures revealed the differences of the in-plane magnetization components. As a result of MOKE image processing, hysteresis loops were obtained from the magneto-optical intensity for different values of the external magnetic field.

### Micromagnetic simulations

Micromagnetic simulations were performed using the MuMax program^17^ running on a Tesla K40 XL graphics processing unit with 12 GB of memory. This graphics processing unit-accelerated open-source software solves the Landau-Lifshitz equation using a finite-difference discretization. The magnetic interactions are included in the simulations in the form of an effective field. Contributions from the following terms were considered in the calculations: an externally applied field that drives the remagnetization process, the magnetostatic field, the Heisenberg exchange field specified by the material constant *A*_ex_, and the magnetoelastic anisotropy field described by the uniaxial anisotropy approximation model. A quasi-static approach was employed to produce the remagnetization curves and the spatial and the temporal magnetic configurations of the domain structures of the microwire.

The quasi-static simulations were equivalent to a nominal variation of the frequency of the applied magnetic field. The simulated cylindrical microwire had a diameter of 1 μm and a length of 15 μm. To choose the appropriate discretization, we calculated the exchange length^18^ Δ_d_ = (2*A*_ex_/*μ*_0_*M*_S_^2^)^1/2^. Using the material parameters of a saturation magnetization *μ*_0_*M*_S_ = 0.7 T and an exchange stiffness constant *A*_ex_ = 2 × 10^−11^ J/m, we obtained Δ_d_ = 10.0125 nm, and therefore the sample was discretized into cells with the dimensions of 10 nm × 10 nm × 5 nm. Following a previous publication^19^, the magnetostriction constant *λ* was selected as 10^−7^. The magnetoelastic anisotropy *K*_me_ was calculated as 3*λσ*/2, where *σ* is the distribution of the internal stress, which was considered using the uniaxial anisotropy approximation model.
